# Acute Synovitis after Trauma Precedes and is Associated with Osteoarthritis Onset and Progression

**DOI:** 10.7150/ijbs.39015

**Published:** 2020-01-30

**Authors:** Lifan Liao, Shanxing Zhang, Lan Zhao, Xiaofeng Chang, Lin Han, Jian Huang, Di Chen

**Affiliations:** 1 Key Laboratory of Shaanxi Province for Craniofacial Precision Medicine Research, Department of Implant Dentistry, Xi'an Jiaotong University College of Stomatology, Xi'an, Shaanxi, China; 2 Department of Orthopedic Surgery, Rush University Medical Center, Chicago, Illinois, USA; 3 Department of Orthopaedic Surgery, The First Affiliated Hospital of Zhejiang Chinese Medical University, Hangzhou, Zhejiang, China; 4 School of Biomedical Engineering, Science, and Health Systems, Drexel University, Philadelphia, Pennsylvania, USA

**Keywords:** osteoarthritis, cartilage, synovitis, subchondral bone

## Abstract

Osteoarthritis (OA) is a whole-joint disease characterized by cartilage destruction, subchondral bone sclerosis, osteophyte formation, and synovitis. However, it remains unclear which part of the joint undergoes initial pathological changes that drives OA onset and progression. In the present study, we investigated the longitudinal alterations of the entire knee joint using a surgically-induced OA mouse model. Histology analysis showed that synovitis occurred as early as 1 week after destabilization of the medial meniscus (DMM), which preceded the events of cartilage degradation, subchondral sclerosis and osteophyte formation. Importantly, key pro-inflammatory cytokines such as IL-1β, IL-6, TNFα, and Ccl2, major matrix degrading enzymes including *Adamts4*, *Mmp3* and *Mmp13*, as well as nerve growth factor (NGF), all increased significantly in both synovium and articular cartilage. It is notable that the inductions of these factors in synovium are far more extensive than those in articular cartilage. Results from behavioral tests demonstrated that sensitization of knee joint pain developed after 8 weeks, later than histological and molecular changes. In addition, the nanoindentation modulus of the medial tibiae decreased 4 weeks after DMM surgery, simultaneous with histological OA signs, which is also later than appearance of synovitis. Collectively, our data suggested that synovitis precedes and is associated with OA, and thus synovium may be an important target to intervene in OA treatment.

## Introduction

Osteoarthritis (OA) is the most prevalent arthritic disease and a leading cause of disability in the United States 1. Knee OA accounts for more than 80% of the disease's total burden 2 and affects at least 19% of American adults aged 45 years and older 3. While OA is a whole-joint disorder characterized by cartilage loss, subchondral bone thickening, synovial inflammation (synovitis) and meniscus degeneration 4, 5, most available OA studies focus on cartilage degradation. Recently, progress in OA studies began to appreciate that synovium and subchondral bone play important roles in the initiation and progression of OA. For example, magnetic resonance imaging often permits an early detection of synovial hypertrophy and bone marrow lesions, both of which can precede cartilage damage 6. Also, a meta-analysis study reported that sonography detects joint effusion and synovial hypertrophy in knee joints in 51.5% and 41.5% of the observed OA patients, respectively 7. However, it remains unclear regarding which part of the joint first undergoes pathological changes and how these changes contribute to OA onset and progression.

During the development of OA, a milestone event is the progressive loss of articular cartilage. Two major structural components of articular cartilage are collagen and aggrecan, which are targeted by cartilage-degrading enzymes such as matrix metalloproteinase (MMPs) and a disintegrin-like and metallopeptidase with thrombospondin type 1 motif (ADAMTs) 8. For example, both Adamts4 and Adamts5 are responsible for aggrecan degradation 9, and MMP13 is an important marker for chondrocyte hypertrophy and a potent enzyme that targets cartilage matrix, especially type II collagen, for degradation 10. Further, OA is also associated with aberrant upregulation of multiple other factors, including runt related transcription factor 2 (Runx2), interleukin 6 (IL-6), interleukin-1β (IL-1β) and nerve growth factor (NGF). Among them, Runx2 plays an important role in chondrocyte differentiation 11, 12 and OA development, and inhibition of Runx2 in chondrocytes could partially rescue DMM-induced OA-like defects in adult mice 13. Col10a1 is the most specific marker for hypertrophic chondrocytes and prominent in osteoarthritic cartilage 14. Abundant expression of pro-inflammatory cytokines, such as IL-1, IL-6, TNFα, and Ccl2, in inflamed synovium is thought to contribute significantly to OA progression 15. NGF is reported to mediate pain through increasing nociceptor sensitivity or facilitating sensory nerve growth 16, and increased synovial NGF is a feature of knee OA 17, 18. In addition, pro-inflammatory IL-1β and TNFα can stimulate the release of NGF from synovial fibroblasts, and NGF can also modulate the release of TNF 19.

In this study, we performed longitudinal analyses of DMM mouse model, in order to identify the pathological and molecular events during OA pathogenesis. We found that the appearance of synovitis is observed as early as 1 week after surgery, which precedes the occurrence of cartilage degradation, subchondral bone sclerosis and osteophyte formation, as well as significantly increased pain sensitivity and decreased modulus values of nanoindentation. Our gene expression profiling results also showed that inductions of pro-inflammatory cytokines, aggrecanases, matrix metalloproteinases, and NGF are significant in both synovium and articular cartilage at the early stage of OA pathogenesis, and the changes are more dramatic in synovium than in cartilage. Together, our data showed that inflammation of synovium is a leading event during comprehensive pathological changes of OA in the entire joint, suggesting that synovial cells could be targeted for early intervention of OA.

## Materials and Methods

### DMM-induced OA model

DMM surgery was performed on the right knee of mice to induce knee OA in 10-week-old C57BL/6 mice 20. Sham operation was performed by opening and exposing the structures of the right knee and then closing the skin incision without manipulating joint tissues. Before and after the surgery, mice were provided with analgesia (2.5 mg/kg Banamine, i. p. injection) every 24 hours for 72 hours and the sutures were removed 10 days after surgery. The right legs were harvested 3 days, 4, 8 and 12 weeks after surgery, for histologic processing, sectioning and staining. The animal protocol of this study has been approved by the Institutional Animal Care and Use Committee of Rush University Medical Center. All experimental methods and procedures were carried out in accordance with the approved guidelines.

### Micro-computed tomography (μCT)

Prior to histologic processing, formalin-fixed mouse legs were scanned using a Scanco μCT35 scanner (Scanco Medical) with a 55 kVp source and a 145 μAmp current. We scanned the mouse legs at a resolution of 12 μm. Morphometric analysis was performed on 50 slices extending proximally, beginning with the first slice in which the tibia condyles had fully merged. To analyze subchondral bone, we manually performed segmentation from cortical shell on the slices using a contouring tool, and then reconstructed and analyzed the morphometry.

### Histology and immunohistochemistry

Knee joint tissues were fixed in 4% paraformaldehyde for 48 hours, decalcified with formic acid for ten days, dehydrated with graded ethanol, and embedded in paraffin 21. Serial sections (3-μm thick) were cut and stained with Alcian blue/Hematoxylin and Orange G or hematoxylin and eosin for morphologic analysis. Immunohistochemistry (IHC) was performed on the 3-μm thick tissue sections 22. Slides were baked at 60°C overnight, deparaffinized, rehydrated, and washed twice in distilled water for 5 minutes each. The antigen retrieval was performed with antigen unmasking solution (Vector Laboratories, H-3300) in 95°C for 10 minutes. Slides were then quenched in 3% hydrogen peroxide for 10 minutes at room temperature, before they were incubated with 0.5% Triton X-100 (Sigma-Aldrich, 9002-93-1) for 1 hour, and washed with PBS for 3 times and then blocked with Avidin/Biotin Blocking Kit (Invitrogen, 004303). Slides were then washed again with PBS for 3 times and then blocked with the blocking serum at 10% normal goat serum (Vector Laboratories, S-1000) in 1% BSA for 30 minutes at room temperature. Slides were then incubated with primary antibodies against MMP13 (MAB 13424, 1:100 dilution) or ColX (ab58632, 1:1000 dilution) at 4°C overnight. On the second day, secondary biotinylated antibody was added for 30 minutes, followed by incubation with VECTASTAIN Elite ABC HRP Kit (Vector Laboratories, PK-6100) for 30 minutes. Positive staining was detected by ImmPACT DAB Peroxidase (HRP) Substrate (Vector Laboratories, PK-6100). Slides were then counterstained with CAT Hematoxylin (Biocare Medical, CATHE-GL), dehydrated with graded ethanol and cleared with 3 changes of Xylene and then coverslipped.

### Quantitative reverse-transcription polymerase chain reaction (PCR)

Articular cartilage and synovium were harvested from the knee joints that underwent the DMM surgery three days after surgery as described previously 23. Total mRNA was extract with Trizol (Invitrogen Life Technologies, CA, USA). Complementary DNA (cDNA) was synthesized using a qScript cDNA Synthesis kit (Quantabio, MA, USA). Real-time PCR amplification was performed using the specific primers of the genes and a SYBR Green real-time PCR kit (Quantabio, MA, USA). The gene names and sequences for the primers were listed in Table 1. Data were collected from the results of 3 independent mice (n=3).

### Hot Plate Test

The hot-plate test was used to measure pain reaction latencies by placing the animals onto a hot plate maintained at 55°C surrounded by glass squares. Specifically, we clean the top surface of the hot plate with alcohol, and then set the temperature of the hot plate at 55°C. After at least 30 min when the hot plate stabilizes at 55℃, mice were placed onto the hot plate and the timer started. Timing is terminated when any of the behavioral events occur, including licking of hind paw, shaking of hind paw in air, or jumping. The animal must be removed from the hotplate as soon as the timer is stopped. The baseline latency should be about 15 sec, and a time less than 10 sec was discarded and the test would be repeated. A cutoff latency of 30 sec is used to prevent tissue damage. Determinations of behavioral latency were performed twice, at least 5 min apart, and the two values were averaged. After the test, the hot plate were shut off, and the top of the plate were cleaned with alcohol.

### Activity Test

Spontaneous activities of the mice were measured to assess if ongoing pain are with the mice 24, 25. Animals are tested in clean, clear vivarium plastic cages (42 cm x 25 cm x 20 cm) using the Photobeam Activity System (San Diego Instruments, San Diego, CA). The photobeam spacing is one inch and photobeam interruptions are recorded automatically. A set of photobeams was set at foot level to measure ambulation, which detects the movement of the mouse from one photobeam to another), and the upper set of photobeams was set 5 cm above ground to measure rearing, which results in photobeam breaks in the vertical direction. Activities were monitored in a low lit room for a 30-minute period.

### Nanoindentation

Nanoindentation tests were performed as described 26. For preparation of the samples, knee joints were harvested from the mice at the time points of 2, 4, and 8 weeks after surgery. Fresh samples were kept in phosphate buffered saline (PBS, PH=7.4) with protease inhibitors (Pierce Protease Inhibitor Tablets # 88266, Thermo Fisher Scientific, Rockford, IL) at 4°C for less than 24 h prior to testing.

### Statistical analysis

Data are expressed as the mean values ± standard deviation. For the experiments comparing two groups of data, unpaired Student *t*-test was performed. For the data involving multiple groups, one-way analysis of variance (ANOVA) followed by Bonferroni's multiple comparison post-test was performed. P values less than 0.05 were considered significant.

## Results

### Histopathological changes during the course of surgically-induced OA progression

**Figure 1:**
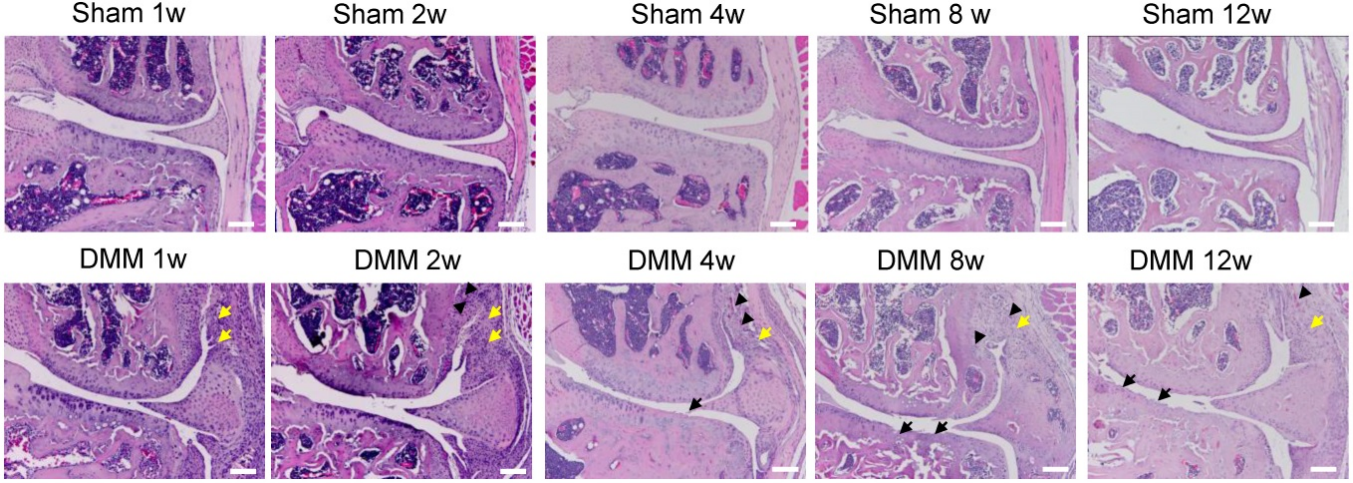
** Synovitis precedes cartilage degradation and angiopoiesis during the pathogenesis of surgically-induced OA.** Knee joints were harvested at different time points of 1, 2, 4, 8, or 12 weeks after surgery as indicated. Hematoxylin-eosin staining results showed synovitis (yellow arrow) in the DMM surgery group one week after surgery, and cartilage degradation (black arrow) and angiopoiesis (black arrowhead) 4 weeks after DMM surgery. Scale bar, 50 μm. n = 5.

**Figure 2:**
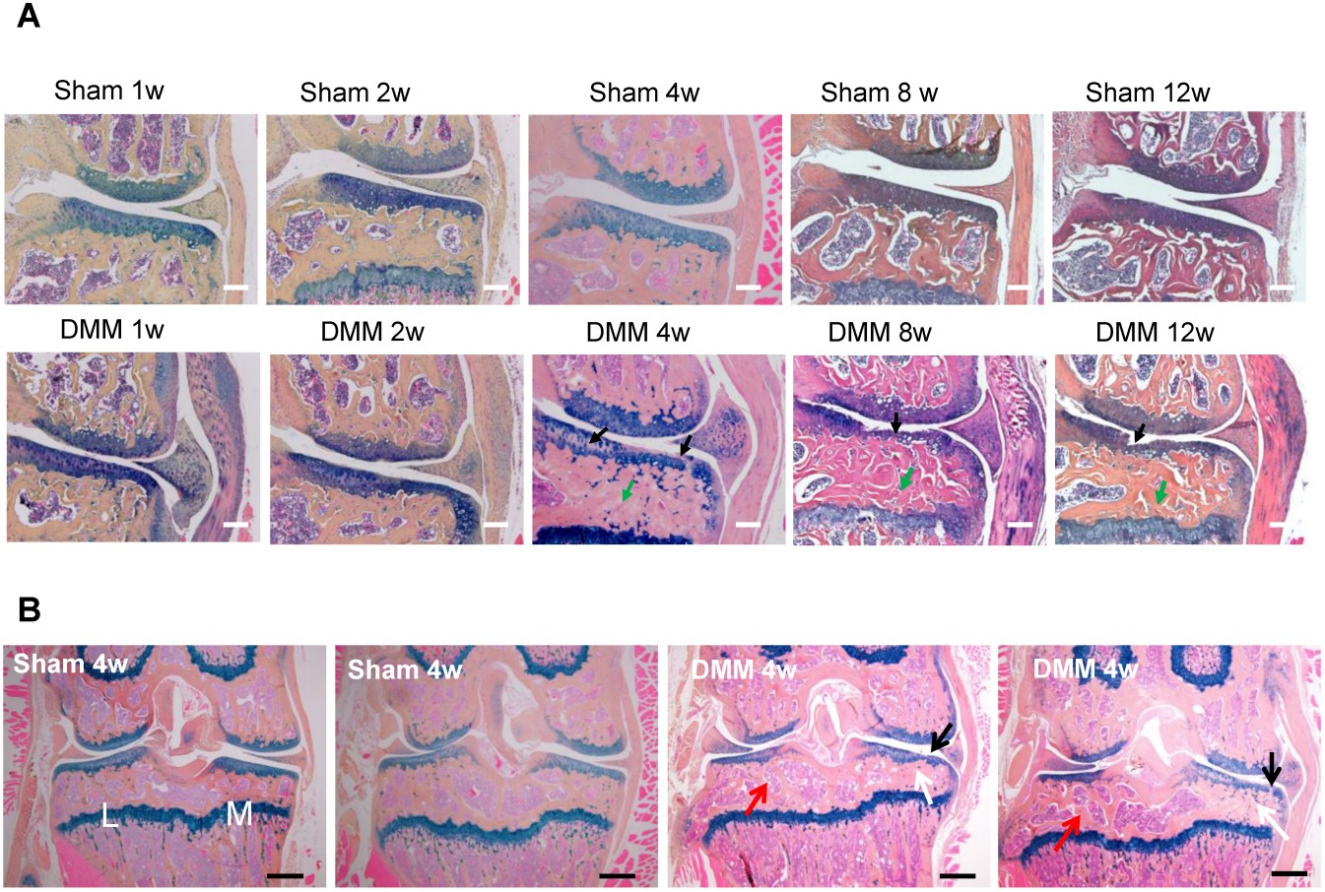
** Longitudinal histologic analysis of cartilage degradation and subchondral bone sclerosis induced by DMM surgery.** (A) Alcian blue/Hematoxylin and Orange G staining of the coronal sections of the knee joints collected at different time points as indicated. Black arrow, cartilage degradation. Green arrow, subchondral bone sclerosis. Scale bar, 50 μm. n = 5. (B) Alcian blue/Hematoxylin Orange G staining of the coronal sections of the joints collected 4 weeks after surgery. Black arrow, cartilage degradation. White arrow, subchondral bone sclerosis in the medial tibiae. Red arrow, subchondral bone loss in the lateral tibiae. L, lateral tibiae; M, medial tibiae. Scale bar, 200 μm. n = 5.

We performed hematoxylin and eosin staining to analyze synovitis in the DMM OA model. One week after the DMM surgery, synovitis was clearly observed in the medial side of the knee joint, as demonstrated by synovial hyperplasia and joint capsule hypertrophy (Figure 1). Cartilage degradation in the medial tibiae and vascular invasion in the medial synovium of the knee joint were already evident 4 weeks after the DMM surgery (Figure 1). We also performed Alcian blue/Hematoxylin and Orange G staining and confirmed cartilage degradation and sclerosis of subchondral bone in the medial tibiae 4 weeks after DMM surgery (Figure 2A). Notably, our data showed that cartilage degradation and subchondral bone sclerosis were observed in the medial tibiae, whereas the loss of subchondral bone was observed in the lateral tibiae at 4w after DMM surgery (Figure 2B). Thus, our results suggested that synovitis could be the first event during the course of surgically-induced OA pathogenesis, which occurs as early as one week after surgery and is followed by the invasion of blood vessels in synovium as well as abrasion and clefts of tibial articular cartilage. Importantly, all of these pathological changes aggravated with time, as histological data at the 8^th^ and 12^th^ week after surgery demonstrated more severe cartilage degradation, synovium hyperplasia and subchondral sclerosis than those at the 4^th^ week (Figure 2A).

### Radiological changes in the knee joints with DMM-induced OA

**Figure 3:**
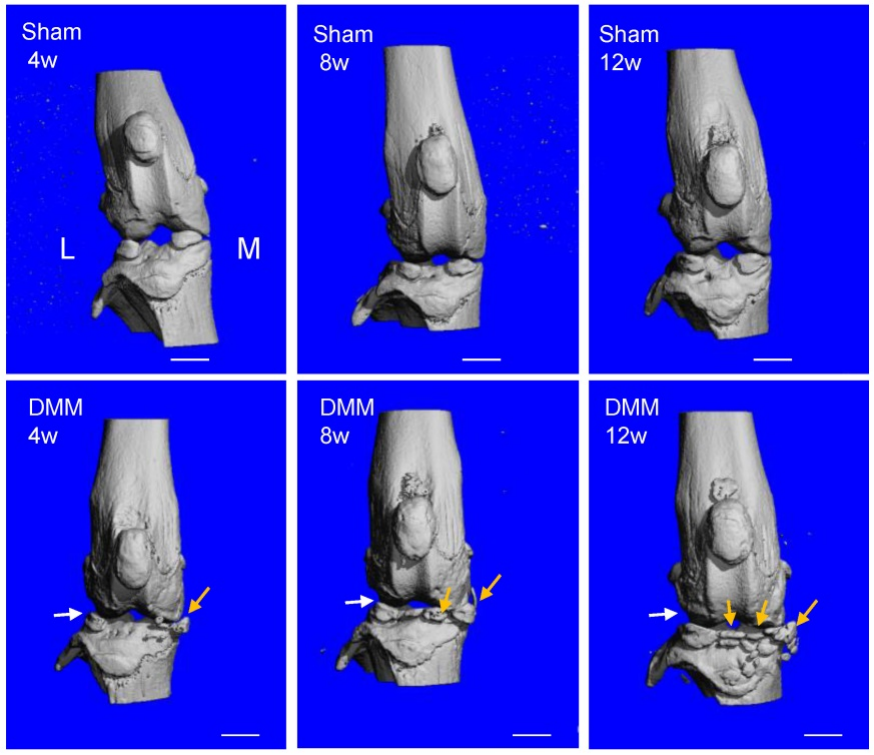
** Longitudinal μCT analysis of osteophyte formation in the pathogenesis of surgically-induced OA.** Osteophytes (yellow arrow) outgrowth and joint space decrease (white arrow) were evident 8 weeks after DMM surgery, and the phenotype aggravated 12 weeks after DMM surgery. L, lateral; M, medial. Scale bar, 1 mm. n = 7.

To evaluate ectopic bone formation induced by the DMM surgery, we performed μCT scanning and analysis of the knee joints. The 3D reconstruction data demonstrated that the joint surface was smooth and the joint space was well-preserved in all the sham groups of 4, 8, and 12 weeks after surgery. In the groups receiving the DMM surgery, minor bony growth in the knee joints initiated 4 weeks after DMM surgery. By referring to the histological staining (Figure 2), we suggested that this bony growth was contributed by the calcification of medial meniscus. Over time, the numbers and volumes of osteophytes increased significantly in the knee joints, as shown by the μCT data of 8 and 12 weeks after DMM surgery (Figure 3). Moreover, clusters of osteophytes occupied the joint space at the time point of 12 weeks, narrowing the joint space significantly (Figure 3). Because synovitis and cartilage degradation are already evident at the time point of 4 weeks after the surgery, our results suggested that the DMM surgery induced meniscus calcification around the fourth week and caused significant osteophyte formation at later time points, which are later events than synovitis and cartilage degradation. Further, our data suggested that the bony changes of osteoarthritic joints are stimulated by other pathological changes in the joint including synovitis and cartilage destruction.

### Alterations of matrix protein and protease levels induced by OA

**Figure 4:**
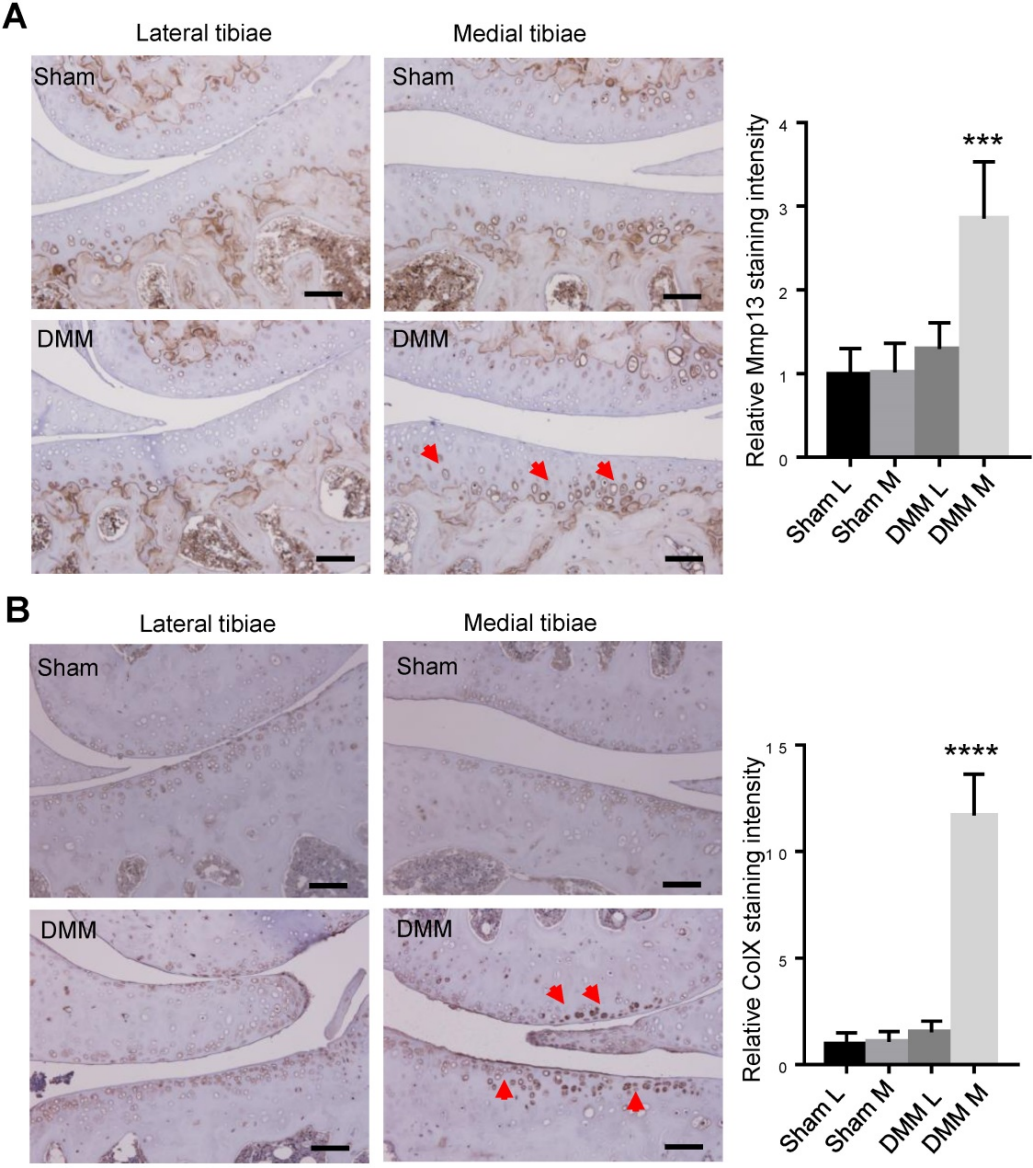
** Inductions of MMP13 and ColX in medial articular cartilage during OA progression.** Immunohistochemistry of MMP13 (A) and ColX (B) of the knee joint sections 4 weeks after DMM surgery. Red arrow, positive IHC signals. It is notable that, in both lateral and medial tibiae of the sham group as well as in the lateral tibiae of the DMM group, MMP13 expressions were weak in articular cartilage and was restricted mainly in deep zone and adjacent to subchondral bone. However, MMP13 expressions increased in medial tibia cartilage and expanded into the middle and superficial zones in the DMM group. Scale bar, 50 μm. *** p < 0.001. **** p < 0.0001. One-way ANOVA followed by Tukey's multiple comparison post-test n = 5.

We had found that histologic changes become evident 8 weeks after DMM surgery 13. To trace earlier changes of cartilage matrix proteins, we harvested the right knee joints at the time point of 4 weeks after surgery for immunohistochemistry (IHC) analysis. Since both MMP13 and ColX are the most important markers of hypertrophic chondrocytes, we preformed IHC to analyze the expression of the two makers 4 weeks after DMM surgery. Our results of immunostaining showed the increased and expanded expression of MMP13 in the middle and superficial zones of medial tibia cartilage (Figure 4A), as well as upregulated expression of ColX in the medial tibia and femur cartilage 4 weeks after DMM surgery (Figure 4B)**,** suggesting that chondrocyte hypertrophy and cartilage degeneration occur before the joint shows evident histologic changes.

### Modulus values of medial tibial cartilage decrease from the fourth week after DMM surgery

**Figure 5:**
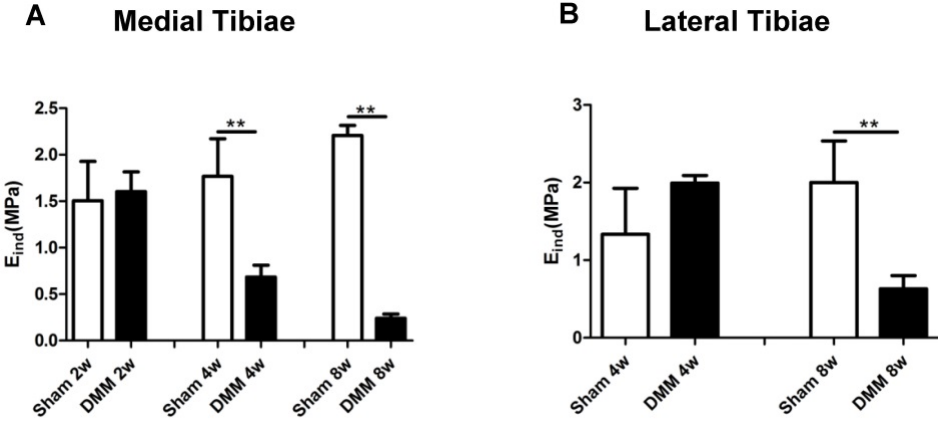
** Long-term changes of nanoindentation modulus of tibia cartilage during OA pathogenesis.** Knee joints were harvested at different time points as indicated for nanoindentation tests. Both medial tibiae (A) and lateral tibiae (B) were measured. * p < 0.05. ** p < 0.01. One-way ANOVA followed by Bonferroni's multiple comparison post-test. n = 4.

Mechanical properties are direct indicators of cartilage function. A previous study reported that nanoindentation modulus of murine cartilage is a sensitive indicator of the initiation and progression of post-traumatic osteoarthritis 27. In our study, modulus values showed no significant difference in both the medial and lateral tibiae at the time point of 2 weeks after DMM surgery, between the sham and the DMM group. However, we detected significant decrease of the nanoindentation modulus values in the medial tibiae 4 weeks after DMM surgery, and this loss of the modulus value aggravated at the time point of 8 weeks after DMM surgery (Figure 5). Notably, in the lateral tibiae, there was no significant difference in the nanoindentation modulus values between the sham and the DMM group 4 weeks after surgery. With OA progression, lateral tibia showed significant decreases of the nanoindentation modulus values 8 weeks after surgery (decreased by 68.5%). Thus, our data suggested that the change of the nanoindentation modulus value in the medial tibiae, i.e. the operated side, occurs earlier than that in the lateral tibiae, which is the non-operated side of the meniscus.

### Longitudinal changes of OA pain-related behaviors

**Figure 6:**
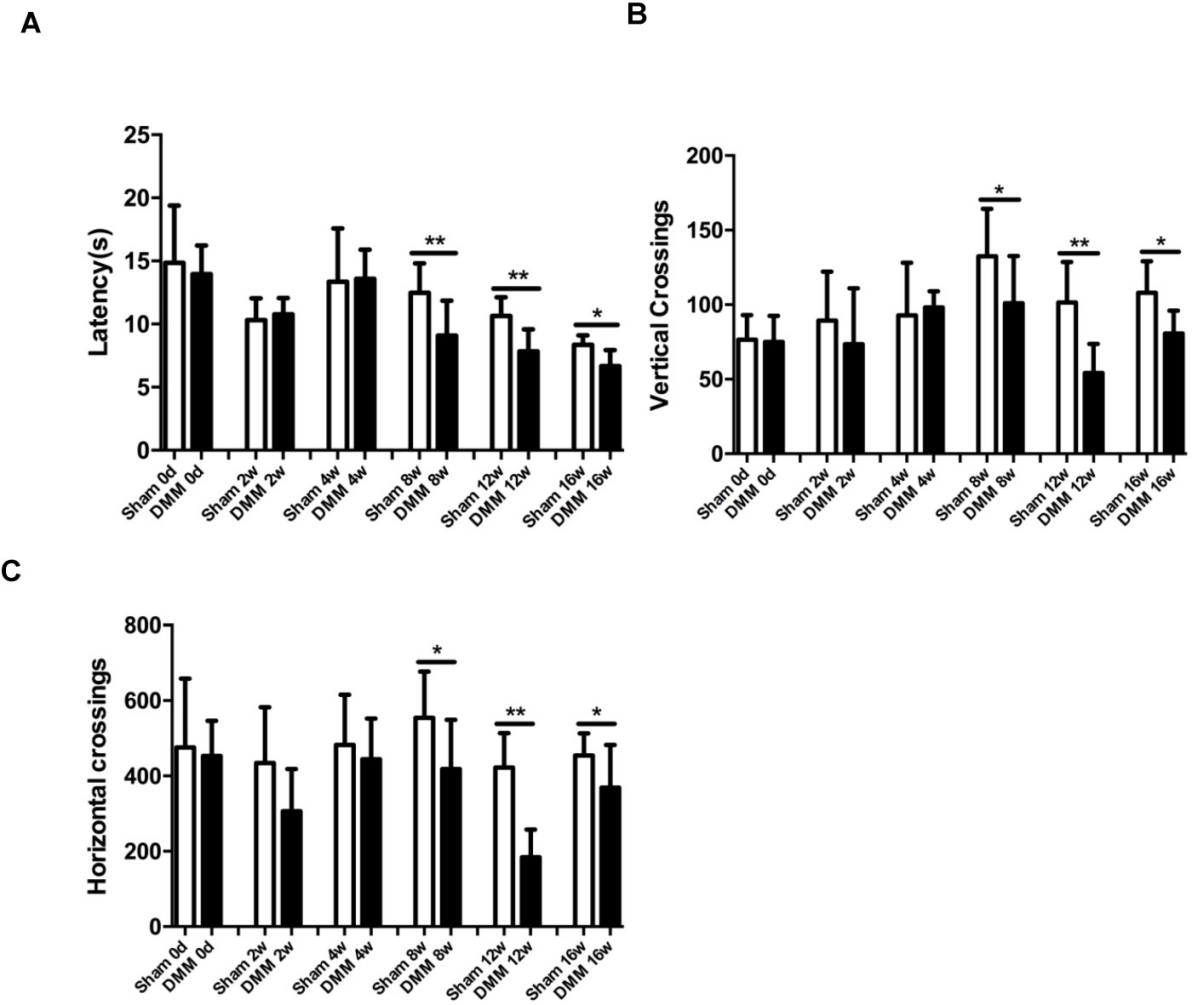
** Longitudinal behavior tests of pain sensitivity induced by DMM surgery. (**A) Hot plate testing results showed that DMM surgery decreased latency since 8 weeks after surgery, suggesting development of thermal hyperalgesia. (B and C) Spontaneous rearing activity, displayed as vertical photobeam crossings (B), and ambulation, measured as horizontal photobeam crossings (C), were reduced since 8 weeks after DMM surgery. * p < 0.05. ** p < 0.01. One-way ANOVA followed Bonferroni's multiple comparison post-test. Values are mean ± SD. n = 7.

One of the hallmark symptoms of osteoarthritis (OA) is pain, which drives individuals to seek medical attention, and contributes to functional limitations and reduced quality of life. To measure OA-related pain in the mice, we performed hot plate tests and spontaneous activity analysis. The data of the hot plate tests showed that, starting from the time points of 8 weeks post DMM surgery, mice with DMM surgery developed significantly lower reaction latencies than mice in the sham group (Figure 6A). Similarly, the results of the spontaneous activity analysis showed no significant difference between the sham group and the DMM group 4 weeks after surgery, but demonstrated significant decrease of both vertical and horizontal movements in the DMM group since the time point of 8 weeks after surgery, suggesting that OA pain became severe with OA progression (Figure 6B and 6C).

### Spatiotemporal profiling of mRNA expressions of OA-associated genes

**Figure 7:**
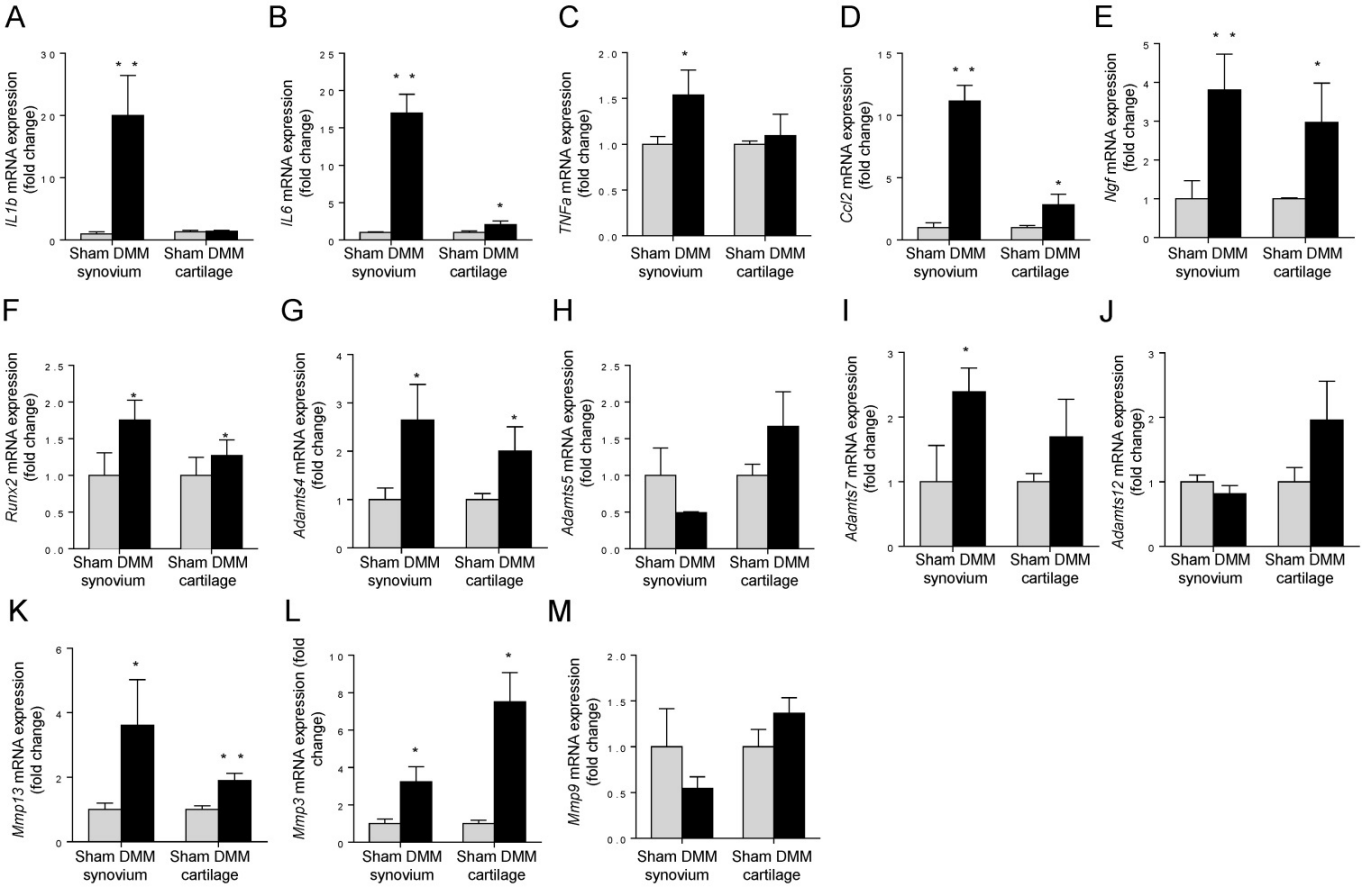
** Early induction of OA-associated factors in synovium.** The mRNA expression levels of inflammatory cytokines (IL-1β, IL-6, TNFα and Ccl2), NGF, Runx2, and extracellular matrix degrading enzymes (Adamts4, Adamts5, Adamts7, Adamts12, Mmp13, Mmp3, and Mmp9) in synovium and articular cartilage were measured 3 days after DMM surgery. * p < 0.05. ** p < 0.01. Unpaired Student's t-test. Values are mean ± SD. n = 3.

To measure the expression changes of OA-associated genes, we isolated total RNA from synovium and tibia cartilage as early as three days after surgery. In the synovium, pro-inflammatory cytokines *IL1b, IL6, TNFα,* and *Ccl2,* increased dramatically in the DMM group compared to those in the sham group (Figure 7A-D). In the tibial cartilage, only the mRNA expressions of *IL6* and* Ccl2* were induced and the extents of the increases were moderate (Figure 7B and 7D), far less than those in the synovium (Figure 7B and 7D). Moreover, the expression of pro-inflammatory cytokines *IL1b* and *TNFα* (Figure 7A and 7C) showed no significant differences compared to those in the sham group in the tibial cartilage (Figure 7A and 7C). NGF, which is related to inflammatory pain through increasing nociceptor sensitivity or facilitate sensory nerve growth, increased significantly in both synovium and cartilage of the DMM group (Figure 7E). In addition, we found that the expressions of *Mmp13* in the synovium and tibial cartilage increased significantly due to the DMM surgery (Figure 7K). As well, Runx2, the important transcription factor that plays a critical role in regulating genes important for chondrocyte hypertrophy and matrix degradation during OA development, increased significantly in both synovium and tibia cartilage three days after DMM surgery (Figure 7F). In summary, our data showed that OA-associated genes are induced in both synovium and cartilage as early as three days after surgery, and the inductions are more prominent in the synovium than in the cartilage, suggesting that the pathological changes of synovium are key events that control OA progression.

## Discussion

OA is the most common joint disorder and a major cause of disability in the adult population 28. It affects a large number of the population. However, currently the molecular etiology of OA is not well-defined and thus there is no cure for it. Murine models offer a unique stepping stone to bridge basic research with clinical practices for OA treatments 29. DMM is now the most commonly used surgical model for OA induction in mice, which is performed by transection of the medial meniscotibial ligament, causing mild instability of the meniscus. Its reliability, reproducibility, phenotypic similarity to human OA, and validity of several pain end points including reversal of pain by standard analgesics, make this model ideal for studying OA pathophysiology. Cartilage destruction is a hallmark and major phenotype of OA. Besides, it is well established that OA is not only a disorder of cartilage homeostasis but also a whole-joint disease involving all structures of the articular tissues, including subchondral bone and synovial membrane 4.

Mechanical properties are direct indicators of cartilage function. Additionally, mechanical changes represent integrated responses of compositional and structural alterations 27, 30. Nanoindentation modulus of murine cartilage is a sensitive indicator of the initiation and progression of post-traumatic osteoarthritis 27. And the reduction of nanoindentation modulus is likely due to concomitantly elevated proteolytic activities 27. In the present study, we have observed that the nanoindentation modulus of the medial tibiae of mice decreased 4 weeks after DMM surgery compared with the sham group, simultaneous with histological OA signs, which became detectable 4 weeks after surgery. Also, we tested the lateral tibiae of knee joints and found that nanoindentation modulus decreased in the DMM group 8 weeks after surgery compared with the sham group, which is later than that in the medial tibiae. Our data suggested that the medial condyle cartilage of tibiae is a more susceptible part to the DMM-related trauma. Nevertheless, lateral condyle cartilage underwent degeneration in later time points, illustrating the whole-organ nature of OA.

Synovitis is now recognized as a characteristic of OA in both its early and late stages, although the degree of inflammation in OA is significantly less than that in RA 31. In the present study, the H&E staining showed obvious inflammation of synovium in the knee joint of the DMM surgery group one week after surgery compared with the sham group, which is earlier than the observed cartilage degradation occurring at 4 weeks after surgery shown by histologic staining and osteophytes appearance at 8 weeks after surgery demonstrated by μCT scanning. Further, our study showed significant inductions of pro-inflammatory cytokines including *IL1b* (18.99 times),* IL6* (15.99 times), *TNFα* (1.54 times), *Ccl2* (10.17 times) in the synovium of the DMM group as early as three days after the DMM surgery. Specifically, these pro-inflammatory cytokines including IL1, IL6 and CCL2 are produced by inflamed synovium and released into the synovial fluid, and may play an essential role in initiating cartilage degradation and promoting OA progression. Collectively, our data suggest that synovitis is an early event in the development of OA.

NGF is reported to mediate inflammatory pain through increasing nociceptor sensitivity or facilitating sensory nerve growth 16. A study reported that increased synovial NGF is a feature of knee OA that may be associated with symptoms 17. In the other hand, pro-inflammatory IL-1β and TNFα can stimulate the release of NGF from synovial fibroblasts, and NGF can also modulate the release of TNF 19. As we found concurrent inductions of *IL1b, TNFα* and *Ngf* in the synovium after DMM surgery, our results may suggest the association between pro-inflammatory cytokines and NGF in the initiation of OA pathogenesis and development of pain-related behavioral changes. In fact, we have observed a significantly increase in pain sensitivity in the DMM group eight weeks after the surgery. Collectively, our results suggest that the molecular and pathological changes in the synovium, including enhanced expression of cytokines and inflamed, hypertrophied synovial tissues, promote NGF expression in the synovium, which then induces sensory nerve growth to cause pathologic alterations of pain-related behavior**.**

Cartilage-degrading enzymes such as Adamts4 and MMP13 play key roles in generating cartilage damage in OA. Multiple studies have demonstrated that synovial macrophages have fundamental effects in positively regulating the production of these proteases, through increasing expressions of pro-inflammatory cytokines. For example, TNFα and/or IL-1β produced by synovial macrophages could drive the expression of *Adamts4* in synovial fibroblasts 32. Also, selective depletion of macrophages in OA cultured synovial cells resulted in downregulation of several fibroblast-produced cytokines and MMPs 33. In our studies, we found significant increases of both *Adamts4* and *Mmp13* in the synovium of the mice as early as 3 days after the DMM surgery. Combined with the data about cytokine induction, our results suggest that *Adamts4* and *Mmp13* may be upregulated by pro-inflammatory cytokines produced by synovial macrophages. In addition, we found Runx2, a transcription factor playing a pivotal role in regulating genes important for chondrocyte hypertrophy and matrix degradation, increased significantly three days after the DMM surgery. Because Runx2 activates *Mmp13* expression by binding to the OSE2 site located in the proximal region of the human *Mmp13* promoter in chondrocytes 34, we concluded that induction of *Mmp13* expression may be also contributed by Runx2 induction in both synovium and tibia cartilage. Together, our results reveal a comprehensive pathway, through which synovial changes induce cartilage degradation at a very early stage, by producing increased levels of cytokines and Runx2 to promote the expression of cartilage catabolic enzymes, initiating cartilage degradation.

As described above, the data from RT-PCR showed that as early as three days after DMM surgery, pro-inflammatory cytokines, aggrecanases, and matrix metalloproteinases, increased significantly in both synovium and tibial cartilage compared to the sham group. However, the increasing extent of these factors in synovium is far more extensive than that in tibia cartilage. It is thought that synovium could influence tibia cartilage and that the influence of the synovium on chondrocytes plays a key role in the pathophysiology of OA 35-37. This is mainly triggered by the release of cytokines and growth factors such as IL-1, IL-6 and TNF 38. These factors are produced by the synovial membrane and diffuse into the cartilage via synovial fluid, which may lead to increased apoptosis of chondrocytes 37. Moreover, a study showed that synovial macrophage activation plays a crucial role in establishing cartilage damage, which suggests that synovitis may be pivotal for OA development 33.

An aim of this project is to find out which part of the knee joint is the chief culprit during the onset of OA. In the present study, the histological changes shown by Alcian blue/Hematoxylin Orange G staining, including the degradation of extracellular matrix and the subchondral sclerosis can be observed at 4 weeks after DMM surgery; additionally, the inflammation of synovial membrane shown by Hematoxylin-eosin staining, can be observed as early as 1 week after DMM surgery. And the osteophytes, another important feature of OA, could be observed by μCT scanning 8 weeks after DMM surgery. Whereas, some molecular changes, such as increased pro-inflammatory related cytokines and extracellular matrix degrading enzymes, could be detected in the synovium of knee joints as early as 3 days after the surgery. Thus these findings clearly suggested the important contribution of synovitis in the onset and development of OA. And the behavioral results establish that DMM surgery sensitizes mice to greater knee joint pain after 8 weeks post induction during OA development, which is later than the events of histological and molecular changes.

Collectively, our results suggest that inflammation of synovium, which occurs prior to cartilage degradation, is an early event during OA initiation and progression. Moreover, inflammatory and destructive responses in the pathogenesis of OA are largely dependent on synovial cells, which produce proinflammatory cytokines such as IL-1β and TNF-α, and therefore have comprehensive effects on the other parts in the joint and contribute to cartilage degradation, synovial hyperplasia, subchondral sclerosis, and OA pain.

## Figures and Tables

**Table 1:** The names and sequences of the primers used in this project

Genes	Primer sequence (forward primers)	Primer sequence (reverse primers)
*IL1b*	GCCTTGGGCCTCAAAGGAAAGAAT	ATTGCTTGGGATCCACACTCTCCA
*IL6*	TGGAGTACCATAGCTACCTGGAGT	CTCTCTGAAGGACTCTGGCTTTGT
*TNFα*	TATGAGCCCATATACCTGGGAGGA	TCCCTTCACAGAGCAATGACTCCA
*Ccl2*	TCACCTGCTGCTACTCATTCACCA	TACAGCTTCTTTGGGACACCTGCT
*Ngf*	ACCACGACTCACACCTTTGTCAAG	CACACACACACAGGCCGTATCTATC
*Runx2*	GACTGTGGTTACCGTCATGGC	ACTTGGTTTTTCATAACAGCGGA
*Mmp3*	TCTTTCACTCAGCCAATGCT	GGGA GGTCCATAGAGGGATT
*Mmp9*	GCAGAGGCATACTTGTACCG	TGATGTTATGATGGTCCCACTTG
*Mmp13*	CTTCTTCTTGTTGAGCTGGACTC	CTGTGGAGGTCACTGTAGACT
*Adamts4*	ATGGCCTCAATCCATCCCAG	GCAAGCAGGGTTGGAATCTTTG
*Adamts5*	GGAGCGAGGCCATTTACAAC	CGTAGACAAGGTAGCCCACTTT
*Adamts7*	GCAGGCTTCGTCTGCTTTCTA	GCCATCAGATAAGGGTTGGTGG
*Adamts12*	GACCCGAGGCAAGAACATTTT	CCCAGTTGACCGTCAGATTGA
*Actin*	GGCTGTATTCCCCTCCATCG	CCAGTTGGTAACAATGCCATGT
